# A Framework to Assist Health Professionals in Recommending High-Quality Apps for Supporting Chronic Disease Self-Management: Illustrative Assessment of Type 2 Diabetes Apps

**DOI:** 10.2196/mhealth.4532

**Published:** 2015-09-14

**Authors:** Kelli Hale, Sandra Capra, Judith Bauer

**Affiliations:** ^1^ Centre of Dietetics Research School of Human Movement and Nutrition Sciences University of Queensland St Lucia Australia

**Keywords:** mobile apps, chronic disease, patient-centered care, technology

## Abstract

**Background:**

This paper presents an approach to assist health professionals in recommending high quality apps for supporting chronic disease self-management. Most app reviews focus on popularity, aesthetics, functionality, usability, and information quality. There is no doubt these factors are important in selecting trustworthy apps which are appealing to users, but behavioral theory may be also be useful in matching the apps to user needs.

**Objective:**

The framework developed aims to be methodologically sound, capable of selecting popular apps which include content covered by evidence-based programs, consistent with behavioral theory, as well as a patient-centered approach for matching apps to patients’ individual needs.

**Methods:**

A single disease—type 2 diabetes—was selected to illustrate how the framework can be applied as this was deemed to represent the types of strategies used in many chronic diseases. A systematic approach based on behavioral theory and recommendations from best practice guidelines was developed for matching apps to patients’ needs. In March 2014, a series of search strategies was used to identify top-rated iPhone and Android health apps, representing 29 topics from five categories of type 2 diabetes self-management strategies. The topics were chosen from published international guidelines for the management of diabetes. The senior author (KH) assessed the most popular apps found that addressed these topics using the Behavioral Theory Content Survey (BTS), which is based on traditional behavioral theory. A tool to assist decision making when using apps was developed and trialed with health professionals for ease of use and understanding.

**Results:**

A total of 14 apps were assessed representing all five topic categories of self-management. Total theoretical scores (BTS scores) were less than 50 on a 100-point scale for all apps. Each app scored less than 50% of the total possible BTS score for all four behavioral theories and for most of the 20 behavioral strategies; however, apps scored higher than 50% of the total possible BTS score for specific strategies related to their primary focus. Our findings suggest that the apps studied would be more effective when used in conjunction with therapy than as stand-alone apps. Apps were categorized according to topic and core intervention strategies. A framework for matching apps to identified patient needs was developed based on app categorization and principles of patient-centered care. The approach was well accepted and understood by a convenience sample of health practitioners.

**Conclusions:**

The framework presented can be used by health practitioners to better match apps with client needs. Some apps incorporate highly interactive strategies of behavioral theory, and when used as an adjunct may increase patient participation and the effectiveness of therapy.

## Introduction

Chronic disease is Australia’s biggest health challenge, accounting for 90% of all deaths in 2011 [1]. These diseases are prolonged in duration, do not often resolve spontaneously, and are rarely cured completely [1]. They are typically associated with lifestyle choices; therefore, for treatment to be effective, patients need to be willing and able to manage their own condition on a daily basis.

Self-management is now considered the appropriate strategy for chronic diseases where lifestyle is critical to management. Traditionally, health professionals have delivered chronic disease self-management (CDSM) interventions to individuals in one-on-one or group situations. Studies have found conventional interventions are most effective when delivered using a patient-centered approach, over long periods, with short follow-up, and regular reinforcement [2]. Unfortunately, these interventions are expensive to implement and difficult to sustain in the primary care setting. Less intensive interventions are needed, and mobile technologies may be helpful as they are affordable and practical. Furthermore, mobile technologies promote increased patient participation which is an essential component of CDSM.

Australians own more advanced-feature mobile phones and have downloaded more apps than many other developed countries. In 2013, 64% of the Australian population owned an advance-feature mobile phone and the average user has 33 apps installed [3]. App use will increase as it is predicted that 91% of the population will own an advanced-feature mobile phone by 2017 [3]. Although app research is limited, many studies have found significant improvements in chronic disease outcomes using mobile interventions [4,5]. Most of the interventions used simple technologies such as short message service (SMS) text messaging for self-monitoring and automated feedback; mobile apps are more sophisticated with real-time, graphic feedback and social functionality.

Due to their popularity, portability, connectivity, and increasing sophistication, apps are an ideal platform for influencing behavior. Despite this, users receive little guidance and support in selecting health apps. Health apps do not require approval from the Therapeutic Goods Administration (TGA) or any other body in Australia to our knowledge. There is a general lack of trust among health professionals in the quality of apps, as many are developed by businesses for commercial gain. A small number of professional organizations recommend apps based on the authenticity of content, user engagement, and aesthetics. While there is no doubt these factors are important in selecting trustworthy apps which are appealing to users, they do not define what apps do or how they can be used to assist in changing behavior.

Current broad international diabetes guidelines recommend interventions be based on behavioral theory [6,7,8]. Behaviorally focused interventions that include interactive strategies have the greatest impact on metabolic and diabetes self-care outcomes [9]. Furthermore, behavioral theories provide a systematic way of explaining and predicting behavior. Social cognitive models have been used as a framework for assessing the behavioral theory content of lifestyle interventions [10,11]. The Behavioral Theory Content Survey (BTS) is a validated tool [10,12] which has been shown to have substantial interrater agreement in assessing mobile apps [12]. It assesses the inclusion and interactivity of 20 intervention strategies which are shared by four key models/theories: (1) Health Belief Model, (2) Theory of Planned Behavior, (3) Transtheoretical Model, and (4) Social Cognitive Theory. While studies have found mobile apps are not usually based on behavioral theory [10,12], many incorporate highly interactive strategies which may support therapy.

This paper presents an approach to assist health professionals in recommending high-quality apps for supporting chronic disease self-management. The framework developed aims to be methodologically sound, capable of selecting popular apps which include content covered by evidence-based programs and consistent with behavioral theory, and a patient-centered approach for matching apps to patients’ individual needs. A single disease—type 2 diabetes—was selected to illustrate how the framework can be applied as this was deemed to represent the types of strategies used in most chronic diseases.

## Methods

### Framework

**Figure 1:**
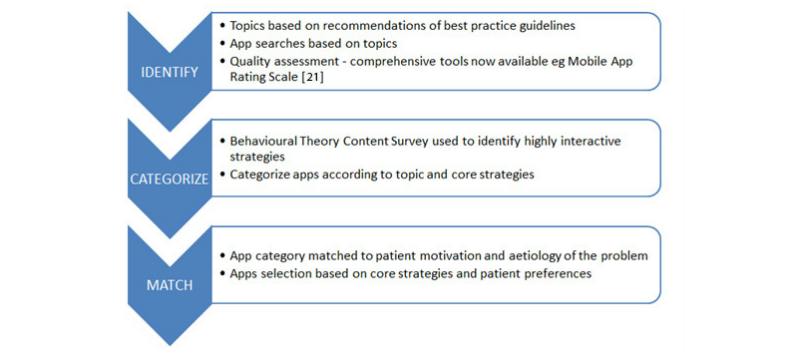
Framework for selecting, categorizing, and matching apps to patients' needs.

We used a three-step process for selecting, categorizing, and matching apps to patients' needs (see Figure 1): (1) identification of popular, high-quality apps which include content covered by evidence-based programs, (2) categorization of apps based on topics and core intervention strategies, and (3) a patient-centered approach for matching apps to patients’ needs.

### App Identification

Using type 2 diabetes as an example in adopting the framework, our aim was to identify popular, high-quality health apps which are consistent with type 2 diabetes evidence-based guidelines.

Apps were selected based on 29 topics identified from the following: (1) patient education topics recommended in the Canadian Diabetes Association 2013 Clinical Practice Guidelines for the Prevention and Management of Diabetes [6] and (2) the seven self-management behaviors identified by the American Association of Diabetes Educators [13]. The topics were grouped into five categories: (1) healthy eating, (2) physical activity, (3) self-monitoring, (4) problem solving, and (5) healthy coping.

A series of search strategies were used to identify eligible health apps available in the Apple App Store and Google Play in March 2014. The apps were first identified from extensive searches of the Apple App Store as it contains the largest number of health apps [14]. Availability was then cross-checked in Google Play. App descriptions and information provided by the Apple App Store were used in the initial screening process. Apps were downloaded to an iPhone and fully explored before selection.

First a search of "Top 200 Free" and "Top 200 Grossing" general and health apps in the Apple App Store was conducted to identify the most downloaded free health apps and the paid health apps generating the most revenue. This was followed by a broad search using the keyword "diabetes." More refined keyword searches followed using keywords specific to topics where less than four apps had been identified in the broader searches including "GI" (glycemic index), "glycemic index," "relaxation," "confidence," and "CBT" (cognitive behavioral therapy). Each selected app was then individually searched in the Google Play Store. Only free and low-cost (ie, less than AUD $5) apps were selected from refined searches as they dominate app purchases [15].

Apps were selected based on the following inclusion criteria: (1) consistent with the 29 app topics, (2) less than AUD $5, and (3) written in English. Apps were excluded if they (1) did not support the International System of Units (SI) of measurements, (2) required extra components to function, (3) were designed specifically for children, (4) did not describe how food databases were compiled, (5) were designed specifically for type 1 diabetes, (6) marketed specific products, and/or (7) contained information that was assessed as inaccurate, biased, or unsafe.

### App Categorization

The apps were grouped according to topic and core intervention strategies. The primary author (KH), having a special interest in health behavior theory, identified the core intervention strategies of each app using the Behaviour Theory Content Survey (see Table 1) [16]. A copy of the evaluation template can be obtained by request from Doshi et al [16].

**Table 1:** Behavioral Theory Content Survey^a^: intervention strategies by behavior change model or theory.

Strategy No.	Intervention strategies^b^	Health Belief Model	Transtheoretical Model	Theory of Planned Behavior	Social Cognitive Theory
1	General information (K)	X	X	X	X
2	Perceived benefits (C)	X	X	X	X
3	Perceived barriers (C)	X	X		X
4	Perceived risks (C)	X		X	
5	Self-efficacy (C)		X	X	X
6	Self-talk (C)		X		X
7	Perceived social norms (C)		X	X	
8	Self-monitoring (B)		X		X
9	Realistic goal setting (B)				X
10	Time management (B)				X
11	Stimulus control (B)		X		X
12	Self-reward (B)		X		X
13	Social support (B)		X		X
14	Modeling/vicarious learning (B)				X
15	Relapse prevention (B)		X		X
16	Stress management (EF)				X
17	Negative affect management (EF)				X
18	Skill building/overview (T)		X		X
19	Increasing knowledge (T)	X	X	X	
20	Motivational readiness (T)		X		
	No. strategies per model/theory	5	14	6	16

^a^With kind permission from Springer Science+Business Media: < Ann Behav Med, Evaluation of physical activity web sites for use of behavior change theories, volume 25, 2003, p107, Doshi A, Patrick K, Sallis JF, et al, Table 1> [16].

^b^Knowledge (K), cognitive (C), behavioral (B), emotion focused (EF), therapeutic (T).

The Behavior Theory Content Survey [16] assesses interventions for the use of 20 strategies (see Table 1) shared by four key behavioral models/theories: (1) Health Belief Model, (2) Theory of Planned Behavior, (3) Transtheoretical Model, and (4) Social Cognitive Theory. The strategies are listed individually as they are common to more than one theory. Each intervention strategy is scored out of 5 as it is rated dichotomously for the inclusion of the following five dimensions of user interaction: (1) provides general information or guidelines, (2) assesses current practices or use of strategies, (3) provides feedback on assessment, (4) offers general assistance on behavior change, and (5) offers individually tailored assistance in response to assessment and feedback. The levels are hierarchical as level 5 (individual advice) is thought to be more effective than level 1 (providing general information). The BTS is the sum of scores for all 20 intervention strategies; the maximum BTS score is 100, representing 20 strategies, each of which are scored out of 5 to indicate the level of interactivity.

### Matching Apps to Patients’ Needs

Health practitioners work with patients (within consultations) to identify core problems and root causes or etiology of problems. Using a patient-centered approach, conventional interventions are usually selected based on problem etiology and patient motivation. We propose that in step 1 apps be selected using the same process, as they are simply another intervention and should complement other therapies. In step 2, the apps are grouped together according to topic and core intervention strategy. Presenting these categories in a table can aid health practitioners in matching apps to the patient’s needs. The primary author (KH) tested the process within dietetic consultations in a primary care setting to determine its usefulness. Next, the tool was further developed based on feedback from a convenience sample of experienced health professionals teaching and examining nutrition therapy.

## Results

### App Identification

Only 4 health apps were recovered from the top 200 free and grossing app searches but they were not specific to diabetes; for example, 2 were exercise apps and 2 were diet-focused apps. Out of the 4 health apps, only 1—clean eating diet app—was ranked in the top 100, and only 2 health apps—exercise apps—met the inclusion criteria.

The most popular free health apps were diet and physical activity apps, and the paid apps generating the most revenue were physical activity and relaxation/meditation apps. The 53 apps which met the eligibility criteria were general diet, physical activity, and relaxation apps. There were no diabetes-specific apps identified in the top or grossing Apple App Store and health app searches.

The 919 apps recovered using the "diabetes" keyword search were a mixture of free and paid apps, including diabetes-specific and general apps. A total of 37 apps met the inclusion criteria and these included diabetes goal-setting, general diet, and general physical activity tracker apps. Fewer than 4 apps were identified from all searches for the topic areas relating to diabetes-specific healthy eating, problem solving, and healthy coping.

An additional 3 apps were recovered using the refined keyword searches, including 2 relaxation apps and 1 CBT app. The search strategies did not recover any eligible diabetes-specific diet apps.

A total of 68 apps were excluded as they were not available on Google Play. A total of 27 apps were available for both iPhone and Android platforms.

### App Categorization

#### App Topics

Apps were identified for all five topics (see Table 2). General apps were relevant to, and could be grouped into, more than one topic. A wider variety of apps were identified for healthy eating and physical activity than for other topic areas.

**Table 2:** Categorization of app types by topic.

Topic categories	App types
Healthy eating	Diet tracker Food selection Menu planning Diabetes-specific goal trackers General goal tracker Coaching Cognitive behavioral therapy
Physical activity	Exercise trackers Resistance exercise Diabetes-specific goal trackers General goal tracker Cognitive behavioral therapy
Self-monitoring	Diabetes tracker Expert support
Problem solving	Coaching Peer support
Healthy coping	Cognitive behavioral therapy Peer support Relaxation

#### Behavioral Theory Content Analysis

Similar apps were grouped together, and only the results for the highest-scoring app for each of the 14 app types are reported in Table 3.

**Table 3:** Total Behavioral Theory Survey score and individual component scores of those apps scoring best in type.

App type	Therapy/model scores^a^				Strategy category scores					Total BTS scores^b^
	HBM^c^	TTM^d^	TPB^e^	SCT^f^	K^g^	C^h^	B^i^	EF^j^	T^k^	
General goals	3	21	7	29	3	4	22	0	2	31
Exercise tracker	2	21	6	26	1	4	20	1	3	29
Diabetes goals	5	21	9	24	2	5	17	0	5	29
Diet tracker	7	19	9	19	3	5	10	2	5	25
Cognitive behavioral therapy	5	18	6	23	1	10	5	4	4	24
Resistance exercise	7	16	8	19	2	5	10	1	5	23
Mindfulness	4	14	5	21	1	5	7	6	3	22
Coaching	7	17	5	20	2	7	7	2	3	21
Menu planning	4	13	4	18	1	3	12	0	3	19
Peer support	2	9	5	12	0	4	9	2	1	16
Expert support	2	12	3	14	1	1	9	1	3	15
Food choice	7	11	8	9	4	2	1	0	4	11
Diabetes tracker	0	9	3	9	0	3	8	0	0	11
Relaxation	1	4	2	9	0	1	1	5	3	10
Mean score (SD)	4.0 (2.4)	14.6 (5.3)	5.7 (2.3)	18.0 (6.5)	1.5 (1.2)	4.2 (2.4)	9.9 (6.2)	1.7 (2.0)	3.1 (1.5)	20.4 (7.0)

^a^Scores do not add up to 100 as the 20 strategies can map to more than one model/theory.

^b^The Behavioral Theory Survey (BTS) score only counts each strategy once and therefore is the sum of the strategy categories; maximum BTS score is 100.

^c^Health Belief Model (HBM); maximum score is 25.

^d^Transtheoretical Model (TTM); maximum score is 70.

^e^Theory of Planned Behavior (TPB); maximum score is 30.

^f^Social Cognitive Theory (SCT); maximum score is 80.

^g^Knowledge (K); maximum score is 5.

^h^Cognitive (C); maximum score is 30.

^i^Behavioral (B); maximum score is 40.

^j^Emotion focused (EF); maximum score is 10.

^k^Therapeutic (T); maximum score is 15.

#### App Scores

The total BTS scores are shown in the last column of Table 3. The mean total BTS score was 20.4 (SD 7.0) out of 100. Apps more often included behavioral (mean 9.9/40, SD 6.2) and knowledge strategies (mean 1.5/5, SD 1.2), and less often used cognitive (mean 4.2/30, SD 2.4) and emotion-focused strategies (mean 1.7/10, SD 2.0). Mean scores for all behavioral models/theories were less than 25% of the total possible scores; scores were highest for Social Cognitive Theory (mean 18.0/80, SD 6.5).

Most apps (11/14, 79%) incorporated more than 50% of the different intervention strategies, but within each strategy, scores were generally less than 2 out of 5. However, all apps included at least one strategy (mean 2.3, SD 1.1) that scored higher than 2 out of 5. Highly interactive intervention strategies, including self-monitoring, social support, modelling/vicarious learning, and stimulus control, were those most commonly included (see Table 4).

**Table 4:** Intervention strategy distribution by all apps.

Intervention strategy	Intervention category	Apps using strategy, n	Apps with BTS^a^>2/5^b^, n
Self-monitoring	Behavioral	12	6
Skill building/overview	Therapeutic	12	2
Social support	Behavioral	10	4
Modeling/vicarious learning	Behavioral	10	5
General information	Knowledge	11	3
Stimulus control	Behavioral	11	5
Realistic goal setting	Behavioral	10	2
Self-efficacy	Cognitive	14	0
Increasing knowledge	Therapeutic	11	1
Negative affect management	Emotion focused	9	2
Perceived social norms	Cognitive	7	0
Perceived barriers	Cognitive	6	1
Time management	Behavioral	6	1
Stress management	Emotion focused	5	2
Self-talk	Cognitive	2	1
Perceived benefits	Cognitive	6	1
Perceived risks	Cognitive	5	0
Self-reward	Behavioral	3	0
Relapse prevention	Behavioral	2	0
Motivational readiness	Therapeutic	1	0

^a^Behavioral Theory Survey (BTS).

^b^Number of apps that scored >2 out of 5 for the intervention strategy. A score above 2 indicates tailored advice or assistance.

### Matching Apps to Patients’ Needs

Table 5 shows how app categorization can be used to assist practitioners in matching apps to patients’ needs.

**Table 5:** Matching apps using type 2 diabetes as an example.

Topic and intervention category: selection based on problem etiology and patient motivation identified during usual practice		App type and core intervention strategies: selection based on patient preference identified during usual practice	
Topic category	Intervention category	App type	Core intervention strategies^a^
Healthy eating	Knowledge	Diet tracker	General knowledge
		Food selection	General knowledge
	Cognitive	Coaching	Perceived barriers
		Cognitive behavioral therapy	Self-talk
	Behavioral	Diet tracker	Self-monitoring
		General goal tracker	Self-monitoring Realistic goal setting Social support Modeling/vicarious learning
		Menu planning	Time management
		Diabetes-specific goal tracker	Self-monitoring Realistic goal setting Social support Modeling/vicarious learning
		Coaching	Stimulus control
	Emotion focused	Cognitive behavioral therapy	Negative affect management
Physical activity	Cognitive	Cognitive behavioral therapy	Self-talk
	Behavioral	Exercise tracker	Self-monitoring Social support Modeling/vicarious learning
		Resistance exercise	Self-monitoring Modelling/vicarious learning
		Diabetes-specific goal tracker	Self-monitoring Realistic goal setting Social support Modeling/vicarious learning
		General goal tracker	Self-monitoring Realistic goal setting Social support Modeling/vicarious learning
	Emotion focused	Cognitive behavioral therapy	Negative affect management
	Therapeutic	Resistance exercise	Skill building/overview
Self-monitoring	Behavioral	Diabetes tracker	Self-monitoring
		Expert support	Stimulus control
Problem solving	Cognitive	Coaching	Perceived barriers
	Behavioral	Peer support	Social support Modeling/vicarious learning
		Coaching	Stimulus control
Healthy coping	Cognitive	Cognitive behavioral therapy	Self-talk
	Behavioral	Peer support	Social support Modeling/vicarious learning
		Mindfulness	Stimulus control
	Emotion focused	Cognitive behavioral therapy	Negative affect management
		Relaxation	Stress management Negative affect management
		Mindfulness	Stress management Negative affect management
	Therapeutic	Cognitive behavioral therapy	Skill building/overview
		Mindfulness	Skill building/overview

^a^Strategies with Behavioral Theory Survey Scores of >2/5. A score above 2 indicates tailored advice or assistance.

## Discussion

### Principal Findings

In this paper we proposed a framework to assist health professionals in recommending high-quality apps for supporting chronic disease self-management. We used type 2 diabetes to illustrate the processes used in (1) creating the app library, (2) identifying core intervention strategies incorporated into apps, and (3) a patient-centered approach to match apps to patient needs.

Our library included apps that incorporated highly interactive strategies from all of the intervention categories. This is different from other studies where the primary focus of the mobile interventions was self-monitoring [4,10,17]. For example, Azar et al [10] found that weight-management apps incorporated mostly behavioral and knowledge strategies and did not use emotion-focused strategies. We selected the most popular apps for topics based on the recommendations from published international guidelines for the management of diabetes, whereas Azar et al [10] searched for a specific type of app (tracker) and included the most popular. Using our search strategy we were able to recover apps that specifically focused on emotion-focused and cognitive strategies.

The behavioral content analysis revealed that most of the apps (11/14, 78%) included more than 50% of theoretical strategies, but total BTS scores were low as few of the highly personalized interactive strategies were included. Apps mostly provided general information and general assistance to users with limited assessment, feedback, or tailored assistance. While apps scored poorly overall, they tended to score high for specific strategies related to their primary focus. Higher-scoring strategies, such as self-monitoring, goal setting, and social support, are associated with healthy eating, higher dietary self-efficacy [18], and Social Cognitive Theory which has been used extensively to explain dietary behavior.

Behavioral theories such as SCT indicate that stand-alone apps would need to use specific combinations of high-scoring strategies to be effective. Table 3 illustrates that all of the apps scored less than 50% of the total possible BTS scores for all four of the behavioral models/theories. The results suggest that even when apps incorporate highly interactive intervention strategies, they cannot replace human factors such as empathy and understanding as they seem not to incorporate sufficient emotion-focused and cognitive strategies. It is as yet unclear if apps that incorporate many strategies would be effective. The general apps would be relevant for a range of chronic diseases. The low scores indicate that a mixture of apps using complementary strategies or apps used in conjunction with more highly interactive interventions would be more effective than solitary apps. Other studies have found mobile interventions are most effective when used as an adjunct to therapy [17,19].

Mobile apps may support and reinforce many aspects of therapy. Tracker apps track symptoms and behavior and are useful to both the health practitioner and the patient. Patients become more aware of their symptoms and behaviors when using assessment apps and this may increase their participation in decision making. Newer technologies objectively estimate food intake and physical activity, reducing demands on users and reliance on self-report. Strategies and goals for behavior change identified in therapy can be programmed into goal-tracking apps which patients can use to prompt new behaviors and monitor progress between visits. Many apps guide patients when practicing new skills and have functions including reminders and social connectivity, which can be used to stimulate desired behavior. App reminders can shape behavior by prompting new behaviors and reminding the patients of motivations for change at predetermined times. Patients can receive encouragement and emotional support from peers via social connectivity. Mobile apps may allow health practitioners to spend less time on assessment and providing general information, and more time on supporting behavior change.

### Practical Application

When used as an adjunct, high-quality health apps may increase the effectiveness of therapy [17,19]. However, patients need guidance from health practitioners for matching apps to their health needs and goals. Table 5 outlines a patient-centered approach for matching apps to patients' needs, preferences, and motivations identified during usual practice. The topic and app category could be based primarily on patient motivation and the etiology of the problem, and the core theoretical strategies of the app could be selected based on patient preference. For example, the diet tracker and/or the food selection app may be the best option(s) for a patient who is motivated to lose weight, wants to focus on diet, and has a food and nutrition knowledge deficit. The diet tracker app will increase the patient’s awareness of how their diet compares to their nutritional goals and will support them in pinpointing less desirable food choices in their diet; the food selection app could be used to identify healthier alternatives.

High-quality apps that are customized to patients’ needs will deliver appropriate guidance, feedback, and triggers for new behaviors, thereby providing intensive support between appointments. It is important that health practitioners provide guidance on how to customize app goals and interpret automated feedback, and provide patients with tailored assistance in further modifying behavior in response to app feedback at follow-up appointments. Empowering patients to use apps should increase their active participation in managing their health. The framework could be used as a basis for future research evaluating the effectiveness of behaviorally based, mobile interventions.

### Strengths and Limitations

The framework presented here is a systematic and methodological approach that was well accepted and understood by a convenience sample of health practitioners. App selection was based on topics recommended in published international guidelines for the management of diabetes, and general criteria focusing on the health practitioners' assessment of information quality and reliability. It uses behavioral theory to explain how apps may be used to support therapy. Studies show mobile text-messaging interventions based on behavioral theory are more effective than non-theory-based ones [20]. Best practice guidelines for chronic disease management of lifestyle-related problems in general recommend basing interventions on behavior change theory. Our framework can be adapted to other conditions, as behavioral theory helps in identifying strategies which match patient needs.

Additionally, the framework can be flexibly delivered to meet practitioners’ needs. For example, some practitioners may not have the time to build the library from scratch, and instead prefer to build it based on their patients’ favorite apps. In this instance, they would skip step one and start by assessing the behavioral theory content of the apps using BTS. This would enable them to advise patients on the best use of preferred apps in supporting behavior change. Using this method, their library will most likely not contain the less popular emotion-focused and cognitive apps. Therefore, regardless of the method, we suggest that these apps be identified using the refined keyword searches described in step 1, for instance, the keywords "GI," "glycemic index," "relaxation," "confidence," and "CBT."

Limitations of the study include the adoption of a relatively general app selection approach that used popularity as a key criterion. Information quality was assessed through professional opinion rather than through a more stringent set of criteria which could not be located at the time. Recently, a comprehensive tool for assessing app quality has been published—the Mobile App Rating Scale [21]. Integration of this tool into the app selection step may increase the quality of the apps included in the library.

### Conclusions

The potential for health apps to support the management of chronic disease is considerable. Health professionals are well positioned to guide patients in the most effective use of apps to meet their needs. Apps are rapidly evolving, so health professionals need to be vigilant and continuously assess apps and refine selection tools for matching apps with therapy. High-quality health apps may be handy instruments for the modern health practitioner’s toolbox.

## Abbreviations

B: behavioral
BTS: Behavioral Theory Content Survey
C: cognitive
CBT: cognitive behavioral therapy
CDSM: chronic disease self-management
EF: emotion focused
GI: glycemic index
HBM: Health Belief Model
K: knowledge
SCT: Social Cognitive Theory
SI: International System of Units
SMS: short message service
T: therapeutic
TGA: Therapeutic Goods Administration
TPB: Theory of Planned Behavior
TTM: Transtheoretical Model
